# Determining Gene Order Patterns in the *Suillus* and Boletales through Comparative Analysis of Their Mitogenomes

**DOI:** 10.3390/ijms25179597

**Published:** 2024-09-04

**Authors:** Jiawei Tao, Xianyi Wang, Yaohang Long, Zexin Gao, Gongyou Zhang, Zhongyao Guo, Guoyu Wang, Guangyin Xu, Yaping Wang, Hongmei Liu

**Affiliations:** 1 School of Public Health, The Key Laboratory of Environmental Pollution Monitoring and Disease Control, Ministry of Education, Guizhou Medical University, Guiyang 561113, China; 2 Engineering Research Center of Medical Biotechnology, School of Biology and Engineering, Guizhou Medical University, Guiyang 561113, China; 3 Engineering Research Center of Health Medicine Biotechnology of Institution of Higher Education of Guizhou Province, Guizhou Medical University, Guiyang 561113, China; 4 Key Laboratory of Biology and Medical Engineering, Immune Cells and Antibody Engineering Research Center of Guizhou Province, School of Biology and Engineering, Guizhou Medical University, Guiyang 561113, China; 5 The High Efficacy Application of Natural Medicinal Resources Engineering Center of Guizhou Province, The Key Laboratory of Optimal Utilization of Natural Medicine Resources, School of Pharmaceutical Sciences, Guizhou Medical University, Guiyang 561113, China; 6 School of Basic Medicine Science, Guizhou Medical University, Guiyang 561113, China

**Keywords:** *Suillus*, gene order pattern, phylogenetic analyses

## Abstract

*Suillus* is one of the most important genera of ectomycorrhizal fungi. As a model for studying host specificity, its molecular fragments and nuclear genome have been analyzed. However, its mitochondrial genome has not yet been reported. In this study, we assembled five mitogenomes of *Suillus* and analyzed and compared their basic characteristics. Owing to the large number of introns as well as intergenic regions, the mitogenomic lengths of species of *Suillus* were greater than those of other species of Boletales. We identified two main patterns of gene order arrangement in the members of the order Boletales. The Ka/Ks values of 15 protein-coding genes were <1 for the mitochondrial genes of 39 Boletales species, indicating their conserved evolution. Phylogenetic trees, reconstructed using the mitogenomes, indicated that the genus *Suillus* was monophyletic. Phylogenetic results based on the internal transcribed spacer region and mitogenome were used to confirm the distribution of *Suillus placidus* in China. The results showed that the mitogenome was superior in distinguishing species compared with a single molecular fragment. This is the first study to investigate the mitogenome of *Suillus*, enriching the mitogenome information and providing basic data for the phylogeny, resource conservation, and genetic diversity of this genus.

## 1. Introduction

The genus *Suillus*, belonging to the family Suillaceae, forms a notable symbiotic relationship with the family Pinaceae. A review suggested that *Suillus* species are one of the ecological drivers of global pine invasions [1] owing to their five main ecological traits: their long-distance dispersal capacity, establishment of positive biotic interactions with mammals, capacity to generate a resistant spore bank, rapid colonization of roots, and long-distance exploration type. Therefore, based on its host specificity patterns and wide distribution, *Suillus* has been widely used to investigate the diversity of ectomycorrhizal fungi and conservation of Pinaceae. In addition, suillin, the ethyl acetate extract of the mushroom *S*. *placidus*, has been found to be an effective therapeutic agent for liver cancer [2]. Some species of *Suillus* are also widely consumed as food items in China [3].

Most species of the genus *Suillus* have a cylindrical, central stipe. The pileus surface may be sticky and slippery when wet, and the cap is usually circular [4]. *Suillus* was originally classified as a genus belonging to the family Boletaceae, but after further study, *Suillus* was assigned to the family Suillaceae, which was mainly established on the basis of chemical data [5]. The genus *Suillus* reportedly comprises three parts: *Paragyrodon*, *Boletinus*, and *Suillus* [6]. However, *Paragyrodon* is currently recognized as belonging to the family Paxillaceae. Researchers have reported a range of *Suillus* species from Asia [3,7,8]. Due to the subjective nature of morphological identification, researchers have also investigated and identified *Suillus* through methods such as chemical systematics and molecular phylogeny [9,10]. Molecular fragment-based phylogenetic studies of *Suillus* first appeared in 1996 [11] and were later supplemented by Nguyen et al. [12]. However, none of these previous studies have focused on a phylogenetic analysis of the mitochondrial genome (mitogenome).

As an independent genetic system outside the nucleus, the mitogenome is characterized by rapid evolution, lack of recombination, high copy number, and maternal inheritance [13]. As a result, it can be used as a new molecular marker and can provide more phylogenetic information than the nuclear gene. Mitogenome sequencing is more accessible and affordable than whole-genome sequencing. With the development of next-generation sequencing (NGS) technology, mitogenomes have been widely employed in the fields of molecular phylogenetics, population genetics, molecular diagnostics, and the geosystematics of fungi [14,15]. Mitogenomes have been used to probe the phylogenetic relationships of the species belonging to the order Boletales, and some comparative analyses of the basic structure and evolution of these mitogenomes have been conducted [16,17,18,19,20,21,22,23]. According to the Macrofungal Taxonomic System and Economic Mushrooms (nmdc.cn), there are approximately 107 species of *Suillus* in the world. Nevertheless, the complete mitogenomes of *Suillus* species have not yet been reported. This has greatly hindered the development of molecular systematics research in this genus.

In this study, five *Suillus* species collected from Guizhou and Yunnan Provinces, China, were sequenced: *S*. *bovinus*, *S*. *huapi*, *S*. *placidus*, *S*. *sibiricus*, and *Suillus* sp. This study had three main objectives. First, to describe the mitogenomic composition of the abovementioned *Suillus* species. Second, to compare the basic composition, evolutionary rates, and conserved sequences of these five mitogenomes. Third, to reconstruct the phylogenetic relationships of Boletales using the complete mitogenomes, in order to explore the evolutionary relationships between members of the genus *Suillus* and other Boletales species. This study provides a theoretical basis for the comprehensive understanding of the structural characteristics and evolutionary diversity of the Suillaceae mitogenome, as well as laying a foundation for the elucidation of the biological function and genome evolution of the Suillaceae.

## 2. Results

### 2.1. Features of the Five Suillus Mitogenomes

**Figure 1:**
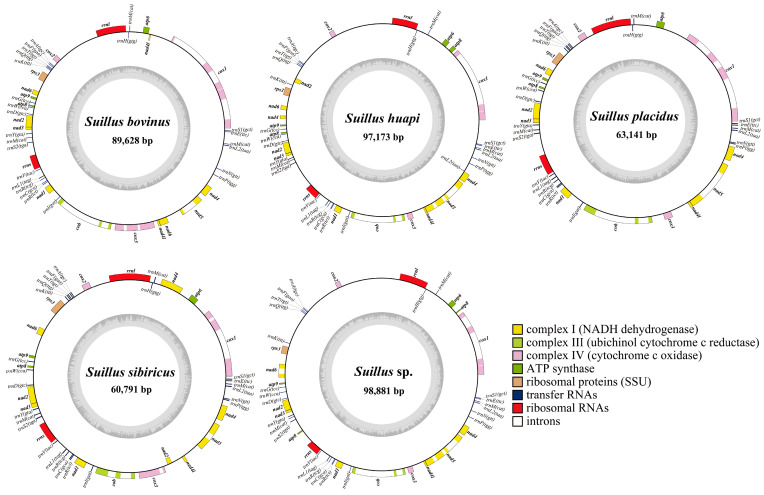
Circular maps of 5 mitogenomes of *Suillus*. Each gene is represented by the corresponding color block. Color blocks outside each ring indicate that the genes are on the direct strand, and color blocks within the ring indicate that the genes are located on the reverse strand.

The complete mitogenomes of the five *Suillus* species were composed of circular DNA molecules (Figure 1). The sizes of these five mitogenomes ranged from 60,791 to 98,881 bp, with an average size of 81,922 bp. *Suillus* sp. contained the largest mitogenome, whereas *S*. *sibiricus* contained the smallest mitogenome (Table 1). All five mitogenomes had a low GC content, ranging from 19.6% to 22.5%, with an average value of 20.76%. All five mitogenomes showed a positive GC skew, ranging from 0.06 to 0.073. The AT skew of the five mitogenomes ranged from −0.024 to 0.006, with an average value of −0.007. All 5 mitogenomes contained 15 PCGs: 1 ribosomal protein S3 (*rps3*), 3 ATP synthase subunits (*atp6*, *atp8*, and *atp9*), 3 cytochrome c oxidase subunits (*cox1*, *cox2*, and *cox3*), 7 NADH dehydrogenase subunits (*nad1*, *nad2*, *nad3*, *nad4*, *nad4l*, *nad5*, and *nad6*), and 1 cytochrome b (*cob*) and 2 rRNAs. All 4 mitogenomes contained 25 transfer RNAs (tRNAs), which encoded the 20 standard amino acids, except for the mitogenome of *S*. *huapi*, which contained 26 tRNAs. This was due to the presence of an extra copy of *trnL2* (TAA). *Dpo*, which is present in the mitogenomes of Boletaceae, was not detected in the mitogenomes of *Suillus*.

### 2.2. PCGs of the Five Suillus Mitogenomes

Among all PCGs, *cox1* showed the highest length in all five *Suillus* mitogenomes, with an average length of 10,902 bp. *S. bovinus* contained the longest *cox1*, while *S*. *sibiricus* contained the shortest *cox1*. *Atp8* was the shortest PCG in all five *Suillus* mitogenomes, with an average length of 176 bp. *S*. *sibiricus*, *S*. *placidus*, and *S. bovinus* contained the longest *atp8*, whereas *S*. *huapi* and *Suillus* sp. contained the shortest *atp8*. The start codons for PCGs in the five mitogenomes included all start codons in the genetic code table 4, except for GTG. Similarly, the termination codons of PCGs in the 5 mitogenomes contained all stop codons in the genetic code table 4, except for TGA. Gene duplication was a frequent occurrence in the mitogenomes of *Suillus*. Duplication of *atp8* occurred in the mitogenomes of *S*. *huapi* and *Suillus* sp.; duplication of *nad6* and *nad4l* occurred in the mitogenome of *S*. *bovinus*; and duplication of *nad4* and *nad2* occurred in the mitogenome of *S*. *sibiricus*. Cytochrome c oxidase showed no duplication in the mitogenomes of *Suillus*.

### 2.3. Gene Rearrangement in the Five Suillus Mitogenomes

**Figure 2:**
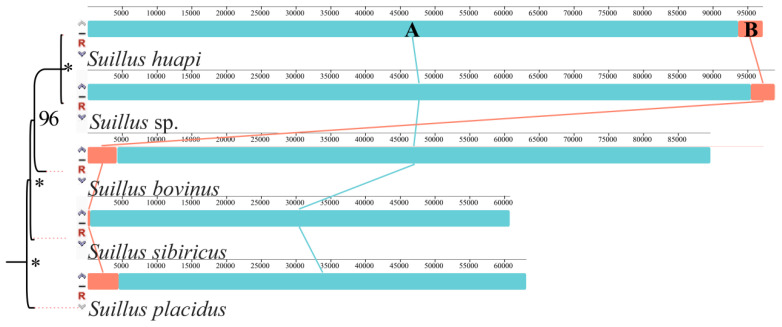
Synteny analysis of the 5 *Suillus* mitogenomes. The symbols * indicate that the posterior probability of this node is 1. Homologous regions between *Suillus* species are color-coded and connected by lines.

After gene synteny analysis of the five *Suillus* mitogenomes, two homologous regions (A and B) were identified (Figure 2). The order of arrangement of regions A and B was the same between *S*. *huapi* and *Suillus* sp. and between *S*. *bovinus*, *S*. *sibiricus*, and *S*. *placidus*. The size of region B in the mitogenome of *S*. *sibiricus* was significantly smaller than that in the remaining four mitogenomes. This may be due to the fact that the mitogenome of *S*. *sibiricus* was also the smallest among the five species. Overall, the results of synteny analysis indicated that *Suillus* evolved conservatively. However, currently, there is insufficient mitogenomic data from *Suillus*, highlighting the need for further research.

**Figure 3:**
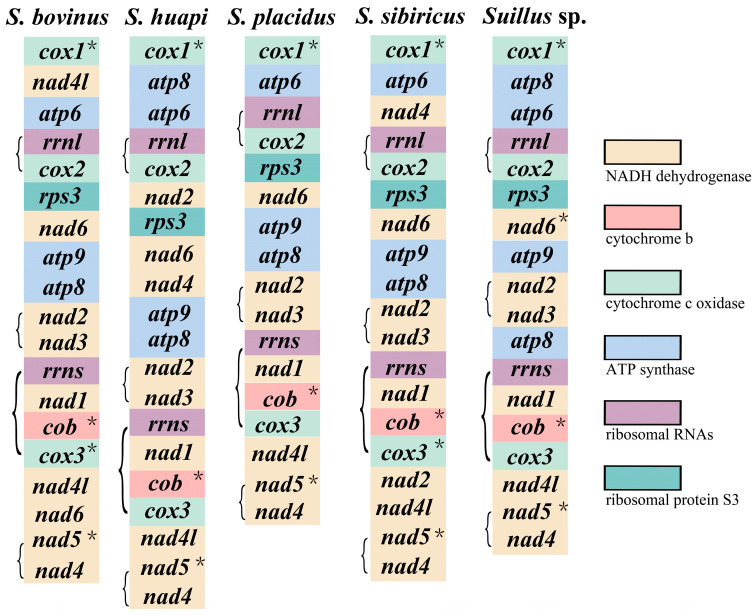
Gene order analysis of *Suillus*. The symbols * indicate that this gene contains introns. Each colored block represents a distinct gene type. 15 PCGs and 2 rRNAs were utilized for analysis.

The gene order of the five *Suillus* mitogenomes was more complex than the results of the synteny analysis suggested (Figure 3). This was probably caused by the duplication and insertion of genes. Four conserved gene clusters were detected in five *Suillus* mitogenomes: *rrnl*-*cox2*, *nad2*-*nad3*, *rrns*-*nad1*-*cob*-*cox3*, and *nad5*-*nad4*. *Rrnl* and *cox2* were in conserved gene order despite non-overlapping regions and long spacer regions. One to four bases overlapped between *nad2* and *nad3*. The number of introns in the mitogenomes of *Suillus* was significantly high. These introns were predominantly found in *cox1*, *cob*, *cox3*, and *nad5*. Furthermore, one intron was identified in *nad6* of *Suillus* sp.

### 2.4. Phylogenetic Relationships of Boletales

**Figure 4:**
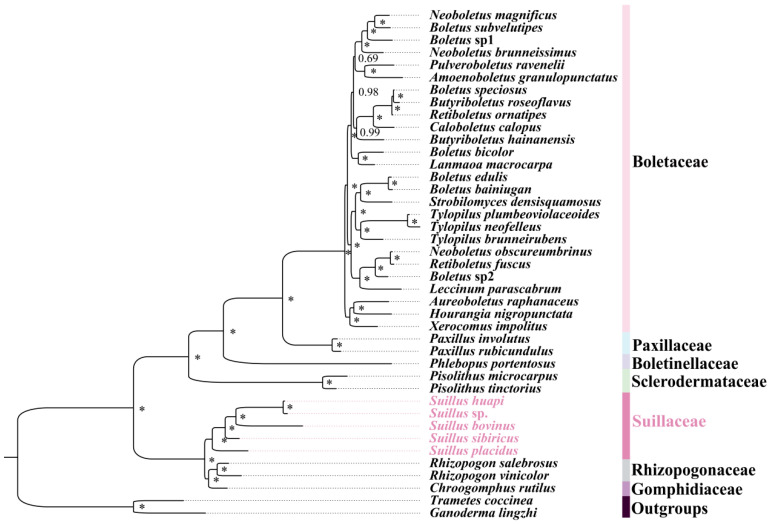
Phylogenetic relationship of Boletales using MrBayes. The symbols * indicate that the posterior probability of this node is 1. Five newly sequenced mitogenomes of *Suillus* highlighted in lavender.

The application of two methods (maximum likelihood, ML, and Bayesian inference, BI) on the 5 datasets (PCG, PCG12, PCGRNA, PCG12RNA, and AA) resulted in nearly identical outcomes. Boletales can be divided into 7 main branches: Boletaceae, Paxillaceae, Boletinellaceae, Sclerodermataceae, Suillaceae, Rhizopogonaceae, and Gomphidiaceae (Figure 4, Figures S1–S9). The monophyly of each family was supported, which justified the existing classification system. *Suillus* were obviously monophyletic, suggesting that they evolved from a common ancestor and were more closely related to each other than to any other species. Throughout the course of evolution, *Suillus* and *Rhizopogon* share a close phylogenetic relationship. *Pisolithus* was once considered to belong to *Suillus* but has since been moved out as a separate genus, which was confirmed by our study. Despite their morphological similarities, *Suillus* is more distantly related to the remaining Boletes species than *Pisolithus*. The 3 families, Suillaceae, Rhizopogonaceae, and Gomphidiaceae, belonging to the suborder Suillineae, also showed close affinities in these phylogenetic trees.

### 2.5. Gene Order Analysis of Boletales

**Figure 5:**
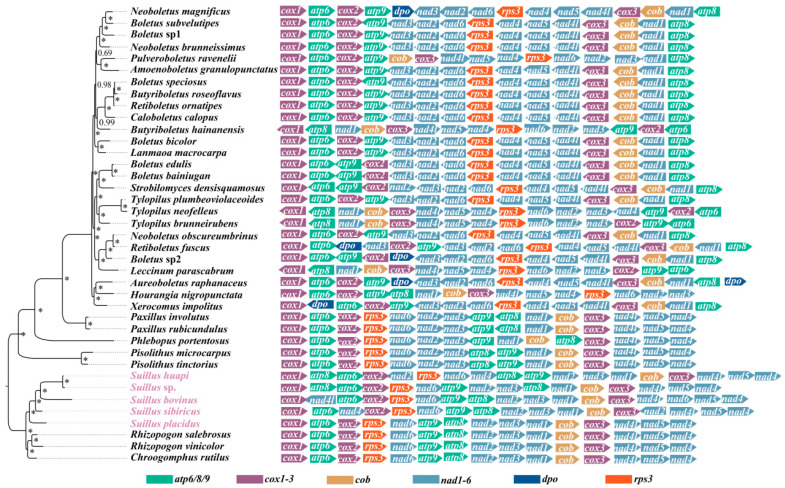
Gene order analysis of Boletales. The symbols * indicate that the posterior probability of this node is 1. Five newly sequenced mitogenomes of *Suillus* were highlighted in lavender. Each colored block represents a distinct gene type. 16 PCGs were utilized for analysis.

To further investigate the associations between gene order and phylogenetic relationships in Boletales, a comparison of mitogenomic gene order was performed for 39 species from the Boletales, covering 20 genera from 7 families. During the evolution of the Boletales, a large number of gene rearrangement events occurred (Figure 5). However, these changes in the location and direction of genes were non-random; there were certain patterns to be found. The orientation of *rps3* was reversed in all but six species, which were in a rather confusing order in Boletaceae. In contrast, in all other families of the Boletales, *rps3* was positively orientated. In the Boletales, *dpo* was only detected in the family Boletaceae, and its position and orientation were relatively random. With the exception of gene order disruption due to the insertion of duplicate genes, the gene order of most species followed a specific pattern (*cox1*-*atp6*-*cox2*-*rps3*-*nad6*-*atp9*-*atp8*-*nad2*-*nad3*-*nad1*-*cob*-*cox3*-*nad4l*-*nad5*-*nad4*) in Suillaceae, Rhizopogonaceae, and Gomphidiaceae. The positions of *atp9*-*atp8* and *nad2*-*nad3* were switched in Paxillaceae and Sclerodermataceae. In contrast, after a certain amount of gene rearrangement as well as phylogenetic evolution, the gene order tended to develop in a pattern (*cox1*-*atp6*-*cox2*-*atp9*-*nad3*-*nad2*-*nad6*-*rps3*- *nad4*-*nad5*-*nad4l*-*cox3*-*cob*-*nad1*-*atp8*) in the Boletaceae.

### 2.6. Evolutionary Rates of Boletales

**Figure 6:**
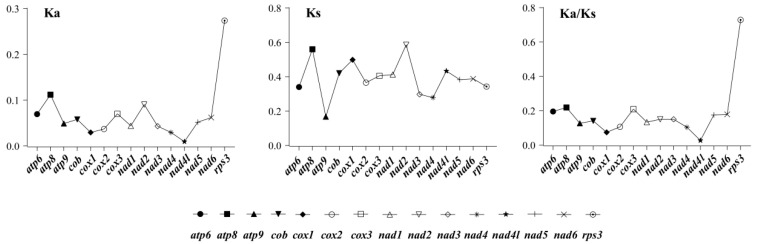
The rate of gene evolution in species of the Boletales. Ka, the number of nonsynonymous substitutions per nonsynonymous site; Ks, the number of synonymous substitutions per synonymous site.

To further explore the evolutionary dynamics of Boletales, PCGs were analyzed for selection pressure at the amino acid level. Due to the skewedness of the data, the median was used to represent the distribution (Figure 6). The nonsynonymous substitution (Ka) rate of *rps3* was significantly higher than that of the other PCGs. Meanwhile, the Ka rate of *nad4l* was the lowest among the 15 PCGs. In general, the Ka values of all 15 genes were found to be less than 0.3. Among these 15 genes, *nad2* had the highest synonymous substitution (Ks) rate, while *atp9* had the lowest. The Ks values of these 15 PCGs were all less than 0.8. The values of Ka/Ks in the 15 PCGs in Boletales were all less than one, and this purifying selection at the amino acid level played a dominant role in each species. *Nad4l* and *cox1* had significantly lower Ka/Ks values, suggesting that these genes had undergone stronger evolutionary selection pressure. Among the 15 PCGs, *rps3* had significantly higher values of Ka/Ks.

## 3. Discussion

Although fungal mitogenomes are intermediate in size between those of animals and plants, they exhibit the greatest variation in size [24]. The size of the fungal mitogenome changes due to several factors, including the accumulation of repetitive sequences, expansion and contraction of introns, and the length of intergenic regions [25,26]. This is true in Boletales, where the presence of introns makes *Suillus* and *Rhizopogon* the two genera with the longest mitogenomes. Interestingly, both *Suillus* and *Rhizopogon* are one of the ecological drivers of global pine invasions [1]. And they are always one of the first fungi to appear on young pine plantations. Perhaps this genomic difference has led to differences in their behavior during the global pine invasions. The length of the intergenic regions is an important factor in the variation in mitogenome length of both Boletaceae and Suillaceae. The variation in mitogenome size within Boletaceae is largely attributed to the accumulation of repetitive sequences. This phenomenon, however, is not observed in the other six families of Boletales.

Although mitochondrial genes are generally conserved owing to their role in cellular metabolism, the order of fungal mitochondrial genes exhibits great variability, which may be attributed to recombination [27]. The mitogenomes of fungi exhibit distinct variations in gene order, both between species and within the same species [28], which was confirmed by the results of this study. The mitogenomic gene order in Boletales species varies considerably between genera and between species of the same genus. Gene duplication causes the highly disorganized gene order in the mitogenomes of *Suillus* species. In these mitogenomes, genes undergo random insertion into a new position after a duplication event, leading to a change in their order. This study broadly categorized the mitogenome gene order of Boletales species into two patterns, consistent with previous research [20]. Intra-molecular recombination of the mitogenome of the common ancestor of the Boletaceae may account for the difference between these two patterns [29]. As a former member of the Agaricales, the PCGs in the mitogenomes of Boletales species exhibited an evolutionary rate that was approximately equivalent to that observed in the mitogenomes of Agaricales species [30]. The Ka/Ks ratios of the 15 PCGs in the mitogenomes of the two orders were less than one, indicating that these genes are still undergoing phylogenetic change.

**Figure 7:**
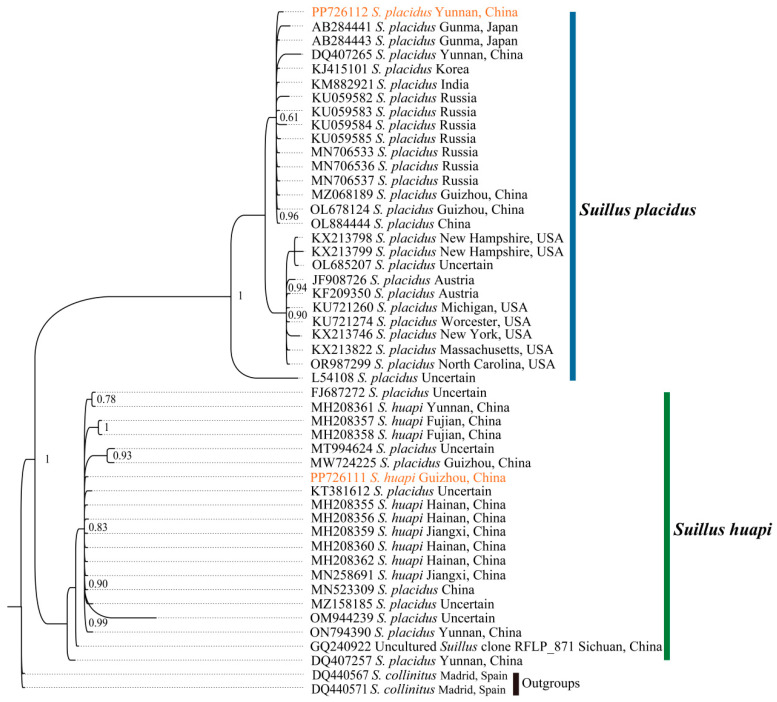
Phylogenetic relationship of *Suillus placidus* and *Suillus huapi* inferred from an *ITS* dataset using Bayesian inference. Our newly sequenced molecular fragments were highlighted in orange.

Previous studies have raised questions regarding the distribution of *Suillus placidus* in China [31]. We concluded that the species previously misidentified as *S. placidus* is actually a well-supported independent lineage, known as *S. huapi*. The two species have distinct physical appearance and ecological characteristics. In *S. huapi*, the color of the pileus changes to brown with growth, whereas in *S. placidus*, it remains white throughout the life cycle. Although all species of *Suillus* form symbiotic relationships with *Pinus* species, the specific species differ. This study provides evidence of the existence of *S. placidus* in China. During our study of the mitogenomes, we observed a clear distinction between *S. placidus* and *S. huapi*. To verify this, *ITS* fragments and the mitogenome were used to distinguish between *S. placidus* and *S. huapi*. The *ITS* was used to reconstruct the phylogenetic tree using the same methods and parameters used in the study by Xue et al. (Figure 7, Figure S10). The molecular fragment reconstruction results indicated a clear division of the *ITS* from the two species into two branches: species *S. placidus* from China exhibited close affinities with species from Asia and Europe. There also appeared to be a clustering of species from the Americas and Australia, while most of the *ITS* of *S. huapi* belonged to Chinese species. Phylogenetic trees inferred from mitogenomes also indicated the presence of *S. placidus* in China. Taken together, the molecular fragments contain less information and are slightly less effective than the mitogenome in distinguishing between easily confused species.

## 4. Materials and Methods

### 4.1. Sample Collection, DNA Isolation, and Sequencing

The five *Suillus* specimens were collected from Guizhou and Yunnan province, China (Table S1). After examination and description, the dried specimens were deposited at Guizhou Medical University. They were first identified by their macroscopic characteristics, such as the pileus, stipe, and hymenophore, and their microscopic characteristics, such as the size of the spores. Then, their identities were confirmed by molecular analysis. DNA samples were extracted from entities of the five specimens using Fungi Genomic DNA Extraction Kits (Solarbio, Beijing, China) and Fungal DNA Kits (Omega, GA, USA), according to the manufacturers’ instructions. The DNA samples were stored at −20 °C until sequencing. The complete mitogenomes of these species were obtained via NGS (Illumina HiSeq 4000 and 6 Gb raw data; Berry Genomic, Beijing, China).

### 4.2. Assembly, Genome Annotation, and Sequence Analysis

The obtained sequences were assembled using Geneious (Available online: https://www.geneious.com, accessed on 5 February–1 March 2024) and compared with homologous sequences of *Aureoboletus raphanaceus* (NC079662) and *Pulveroboletus ravenelii* (NC061666) [16]. They were then identified through BLAST searches in the NCBI database. The mitogenomes of five *Suillus* species were annotated using MITOS based on the Galaxy platform [32] and genetic code table 4 (mold protozoan mitochondrial). The locations of the large ribosomal subunit (*rrnl*) and small ribosomal subunit (*rrns*) were ascertained via comparison with homologous mitogenomes. The locations of 15 protein-coding genes (PCGs) were confirmed using ORF Finder in Geneious. Finally, all genes with reference were compared to verify their validity. Five circular mitogenomic maps were constructed using OGDraw version 1.3.1 [33]. The strand asymmetry was calculated using the following formulas: AT skew = (A − T)/(A + T) and GC skew = (G − C)/(G + C) [34].

Nonsynonymous substitution rates (Ka) and synonymous substitution rates (Ks) of 15 core PCGs (*atp6*, *atp8*, *atp9*, *cox1*, *cox2*, *cox3*, *cob*, *nad1*, *nad2*, *nad3*, *nad4*, *nad4l*, *nad5*, *nad6*, and *rps3*) in the Boletales mitogenomes were calculated using DnaSP version 6.12.03 to explore the genomic evolution of Boletales [35]. Gene synteny analysis of the five *Suillus* mitogenomes was performed using Mauve version 2.4.0 in Geneious [36].

### 4.3. Phylogenetic Analysis

To explore the phylogenetic relationships of the genus *Suillus*, we reconstructed a phylogenetic tree of the order Boletales. A total of 39 complete mitogenomes of Boletales species were included. The complete mitogenomes of two Polyporales species, namely, *Trametes coccinea* and *Ganoderma lingzhi* (Table S2), were used as outgroups [37,38]. MW308606 was labeled as *Boletus* sp1 and MW308608 was labeled as *Boletus* sp2 to distinguish them from each other. The phylogenetic relationships within Boletales were analyzed using the sequences of 15 PCGs (*atp6*, *atp8*, *atp9*, *cox1*, *cox2*, *cox3*, *cob*, *nad1*, *nad2*, *nad3*, *nad4*, *nad4l*, *nad5*, *nad6*, and *rps3*) and 2 rRNAs (*rrnl* and *rrns*). Each PCG and rRNA sequence was aligned using the MAFFT algorithm in Translator X and MAFFT version 7.0 online service with G-INS-i strategy, respectively [39,40]. Poorly aligned sites were removed using Gblocks 0.91b [41] under default settings. Subsequently, the resulting 16 alignments were assessed and manually corrected using MEGA 7 [42]. Five datasets were obtained, namely, PCG (all codon positions of 15 PCGs), PCG12 (first and second codon positions of 15 PCGs), PCGRNA (PCG and two rRNAs), PCG12RNA (PCG12 and two rRNAs), and AA (amino acid sequences of 15 PCGs).

Phylogenetic trees were reconstructed using ML and BI analyses. ML analysis was conducted in IQ-TREE version 1.6.12 with 1000 bootstrap replicates to estimate node support using the ultrafast bootstrap approach [43]. BI trees were constructed using MrBayes v.3.2.7 [44] with the default settings to simulate four independent runs for 1 million generations, with sampling every 1000 generations. After the average standard deviation of split frequencies decreased to <0.01, the initial 25% of the samples were discarded as burn-in, and the remaining trees were used to generate a consensus tree and to calculate the posterior probabilities. Finally, the phylogenetic trees were visualized using FigTree version 1.4.4 (Available online: tree.bio.ed.ac.uk/software/Figtree/, accessed on 7 May 2024) and enhanced using Adobe Illustrator CC version 22.1.

To determine whether *S. placidus* and *S. huapi* were the same species and explore the differences in phylogenetic relationships obtained using mitogenomes and molecular fragments, we chose *ITS* data, a molecular fragment with the most data, for these two species to reconstruct the phylogenetic relationship. All sequences were aligned using the MAFFT algorithm in PhyloSuite [45]. Poorly aligned sites were removed using Gblocks 0.91b., and MrBayes v.3.2.7 was used to construct BI trees. ML analysis was conducted via IQ-TREE. Visualization of phylogenetic trees was performed using FigTree and Adobe Illustrator CC version 22.1.

## 5. Conclusions

*Suillus* is a crucial ectomycorrhizal fungus as well as a valuable edible and medicinal mushroom. Owing to their similar morphology, researchers have conducted studies to differentiate the species of *Suillus* [46,47]. However, there is limited information available on molecular fragments, which hinders the efforts to differentiate species. Mitochondrial genomes are commonly used to differentiate species and phylogeny because of their high copy rate and easy accessibility [48,49]. Studies have reported mitogenomes for many species of Boletales, but not for any species of *Suillus*. In this study, we collected, sequenced, assembled, and annotated the mitogenomes of five *Suillus* species. These five mitogenomes were analyzed comparatively and phylogenetically with those of other species of Boletales. All five mitogenomes were probed for at least 15 PCGs, 25 tRNAs, and 2 ribosomal RNAs. This study revealed that these mitogenomes had common duplications of the ATP synthase subunit and NADH dehydrogenase subunit. The results of gene synteny and gene order analyses were slightly different between the mitogenomes of *Suillus* species. The former suggested that species of *Suillus* were more conserved, while the latter indicated that *Suillus* species were more disordered due to the insertion of duplicate genes. Phylogenetic analysis validated the existing taxonomic system of Boletales species at the family level, with support for the monophyly of the seven families. There were two main patterns of gene order in Boletales, and the insertion of duplicated genes and *dpo* may disrupt the gene order. Analysis of 15 PCGs of Boletales for selection pressure showed that these genes were subject to purifying selection. This study revealed that the mitogenome provides insights into the evolutionary relationships of *Suillus* and Boletales, aids in constructing accurate phylogenetic trees, and serves as a molecular marker for species identification. Further research is warranted to better understand the mitogenomes of *Suillus* species, including their evolution patterns and mechanisms, as well as the relationship between mitogenomes and fungal functions and adaptations.

## Figures and Tables

**Table 1:** Basic composition of 5 new mitogenomes of *Suillus*.

Species	Length (bp)	GC Rate (%)	AT Skew	GC Skew	tRNAs	Introns
*S. bovinus*	89,628	20.7	−0.009	0.060	25	9
*S. huapi*	97,173	19.7	0.006	0.073	26	7
*S. placidus*	63,141	22.5	−0.010	0.067	25	6
*S. sibiricus*	60,791	21.3	−0.024	0.066	25	6
*Suillus* sp.	98,881	19.6	0.002	0.073	25	9

## Data Availability

The five newly sequenced mitogenomes were deposited at GenBank under the following accession numbers: PP727298–PP727302. *S*. *bovinus* (PP727298); *S*. *huapi* (PP727299); *S*. *placidus* (PP727300); *S*. *sibiricus* (PP727301); *Suillus* sp. (PP727302). The two newly sequenced *ITS* were deposited at GenBank under the following accession numbers: PP726111 (*S*. *huapi*) and PP726112 (*S*. *placidus*).

## Supplementary Materials

The following supporting information can be downloaded at: https://www.mdpi.com/article/10.3390/ijms25179597/s1.
