# Supplementary material for: Determining Gene Order Patterns in the Suillus and Boletales through Comparative Analysis of Their Mitogenomes
Source: Int J Mol Sci. 2024 Sep 4;25(17):9597. doi: 10.3390/ijms25179597 (PMC11394714; doi:10.3390/ijms25179597)
Supplement: Supplementary file 1 [file ijms-25-09597-s001.zip › ijms-3183418-supplementary.pdf]

Table S1. Collect information of five *Suillus*.

Species name	Locality	Altitude (m)	Longitude and latitude	Habitat and distribution	Time
S. bovinus	Guiyang City, Guizhou Province	1169.598	106°76′, 26°56′	Gregarious on soils in coniferous forest	2023.6.22
S. huapi	Longli County, Guizhou Province	1205.530	106°92′, 26°43′	Gregarious on soils in two-needled forest	2023.6.18
S. placidus	Songming County, Yunnan Province	2220.335	102°93′, 25°27′	Solitary or gregarious on soils in five-needled forest	2023.7.29
S. sibiricus	Guiyang City, Guizhou Province	1287.641	106°39′, 26°39′	Solitary or gregarious on soils in five-needled forest	2023.5.21
Suillus sp.	Longli County, Guizhou Province	1205.530	106°92′, 26°43′	Solitary on soils in coniferous forest	2023.6.18

Table S2. Information of samples for phylogenetic analysis.

Family	Species	Genbank Number	Size (bp)	References
Boletaceae	*Amoenoboletus granulopunctatus*	PP048753	44,774	Unpublished
	Aureoboletus raphanaceus	NC079662	42,157	Unpublished
	Boletus bainiugan	PP048754	34,893	Unpublished
	Boletus bicolor	MW308599	38,082	[20]
	Boletus edulis	MW308609	34,763	[20]
	Boletus sp.	MW308606	35,304	[20]
	Boletus sp.	MW308608	48,298	[20]
	Boletus speciosus	MW308600	36,622	[20]
	Boletus subvelutipes	MW308604	34,668	[20]
	Butyriboletus hainanensis	NC082970	36,592	Unpublished
	Butyriboletus roseoflavus	MZ202357	36,551	Unpublished
	Caloboletus calopus	MW308602	32,883	[20]
	Hourangia nigropunctata	PP048751	32,910	Unpublished
	Lanmaoa macrocarpa	NC080885	38,139	Unpublished
	Leccinum parascabrum	PP048752	41,354	Unpublished
	Neoboletus brunneissimus	MW308605	42,147	[20]
	Neoboletus magnificus	MW308603	39,449	[20]
	Neoboletus obscureumbrinus	MW308607	39,929	[20]
	Pulveroboletus ravenelii	NC061666	43,528	[16]
	Retiboletus fuscus	PP048756	43,616	Unpublished
	Retiboletus ornatipes	MW308601	36,785	[20]
	Strobilomyces densisquamosus	PP048755	36,132	Unpublished
	Tylopilus brunneirubens	NC084291	32,389	Unpublished
	Tylopilus neofelleus	PP048750	33,453	Unpublished
	Tylopilus plumbeoviolaceoides	NC056835	37,242	[19]
	Xerocomus impolitus	NC056808	39,362	Unpublished
Paxillaceae	*Paxillus involutus*	NC045203	39,109	[22]
	Paxillus rubicundulus	NC045204	41,061	[22]
Boletinellaceae	*Phlebopus portentosus*	MK571437	43,299	[23]
Sclerodermataceae	*Pisolithus microcarpus*	NC054201	43,990	[18]
	Pisolithus tinctorius	NC054202	44,054	[18]
Suillaceae	***S. bovinus***	**PP727298**	**89, 628**	**This study**
	S. huapi	**PP727299**	**97, 173**	**This study**
	S. placidus	**PP727300**	**63, 141**	**This study**
	S. sibiricus	**PP727301**	**60, 791**	**This study**
	***Suillus* sp.**	**PP727302**	**98, 881**	**This study**
Rhizopogonaceae	*Rhizopogon salebrosus*	NC042698	66,704	[21]
	Rhizopogon vinicolor	NC042699	77,109	[21]
Gomphidiaceae	*Chroogomphus rutilus*	MZ151416	37,508	[17]
Outgroups	*Ganoderma lingzhi*	NC062658	56,044	[37]
	Trametes coccinea	NC054272	99,976	[38]

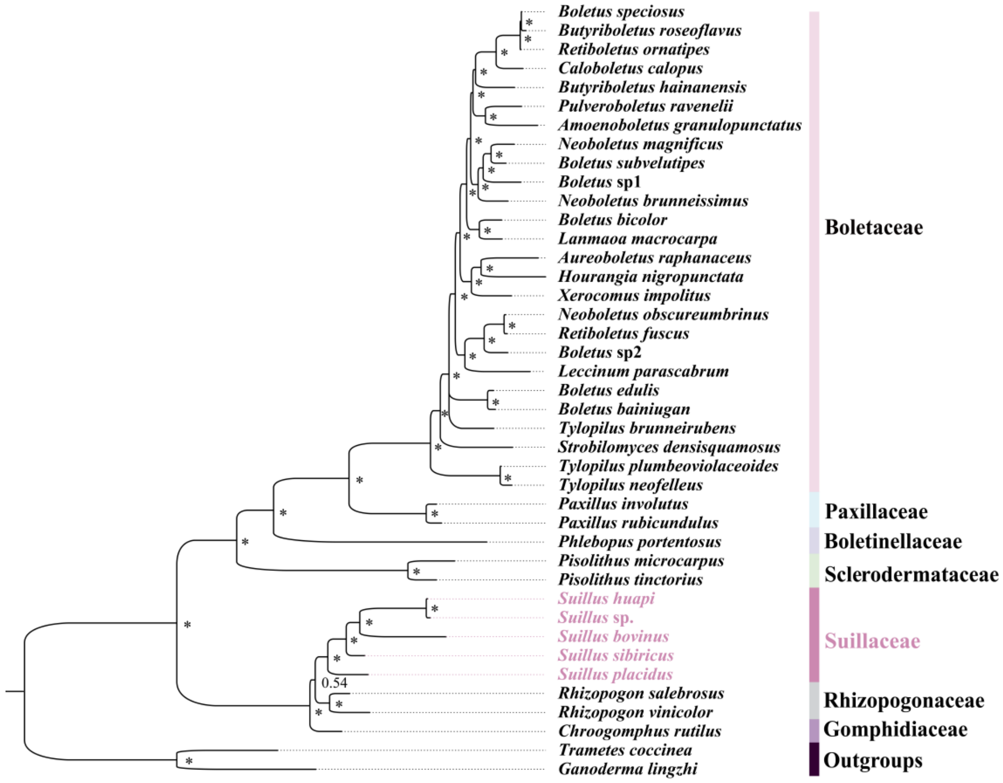

Figure S1. Phylogenetic relationship of Boletales based on PCG using MrBayes. The symbols * indicate that the posterior probability of this node is 1. Five newly sequenced mitogenomes of *Suillus* were highlighted in lavender.

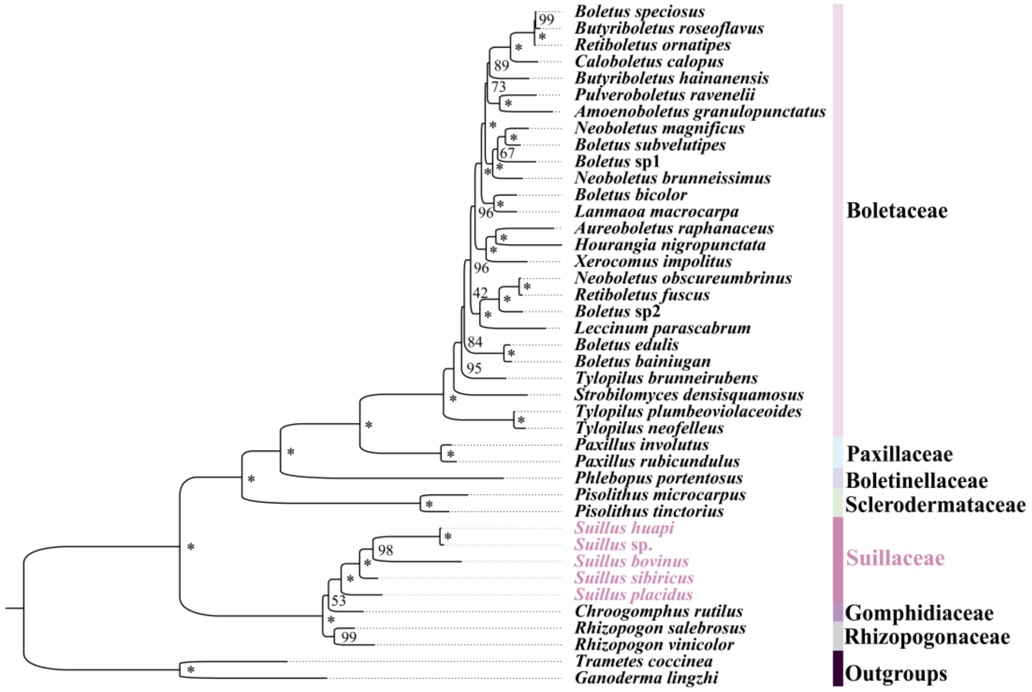

Figure S2. Phylogenetic relationship of Boletales based on PCG using IQ-TREE. The symbols * indicate that BS of this node is 100. Five newly sequenced mitogenomes of *Suillus* were highlighted in lavender.

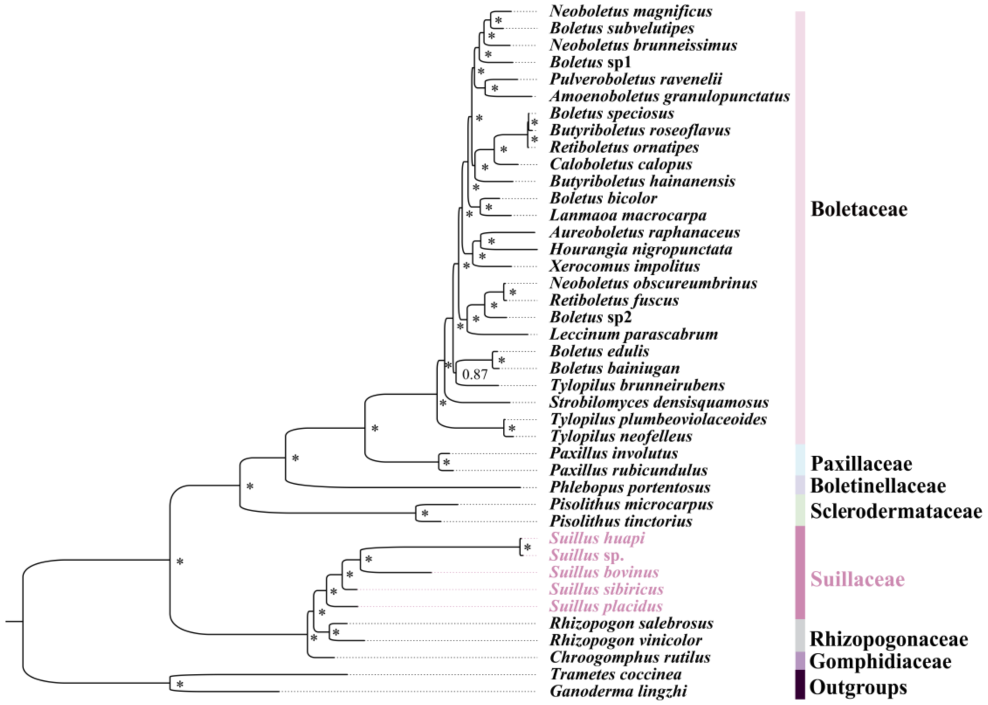

Figure S3. Phylogenetic relationship of Boletales based on PCGRNA using MrBayes. The symbols * indicate that the posterior probability of this node is 1. Five newly sequenced mitogenomes of *Suillus* were highlighted in lavender.

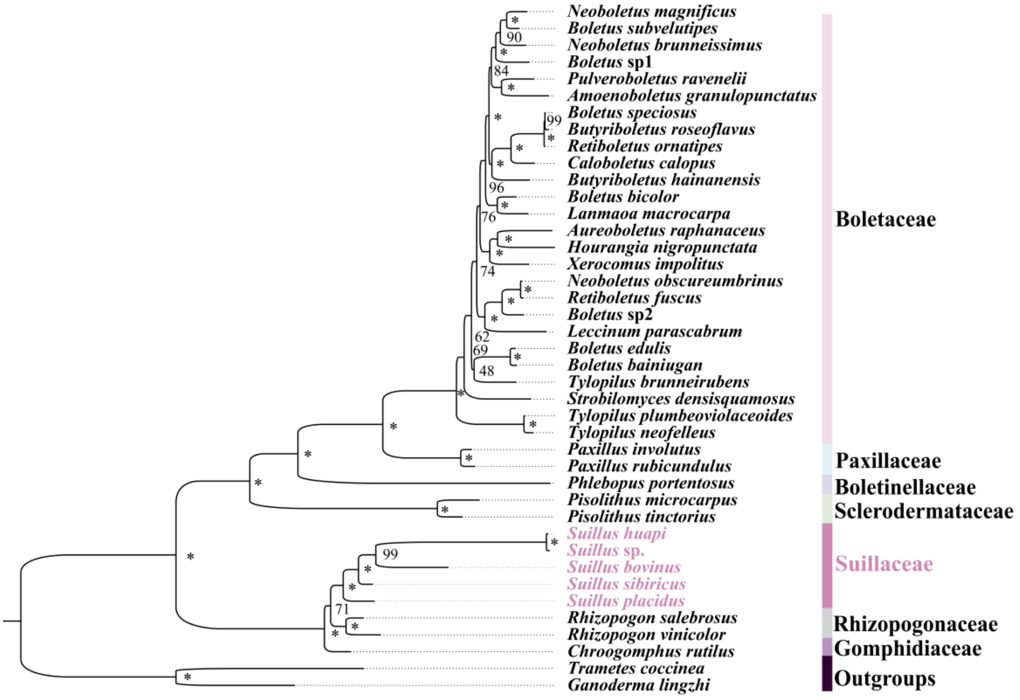

Figure S4. Phylogenetic relationship of Boletales based on PCGRNA using IQ-TREE. The symbols * indicate that BS of this node is 100. Five newly sequenced mitogenomes of *Suillus* were highlighted in lavender.

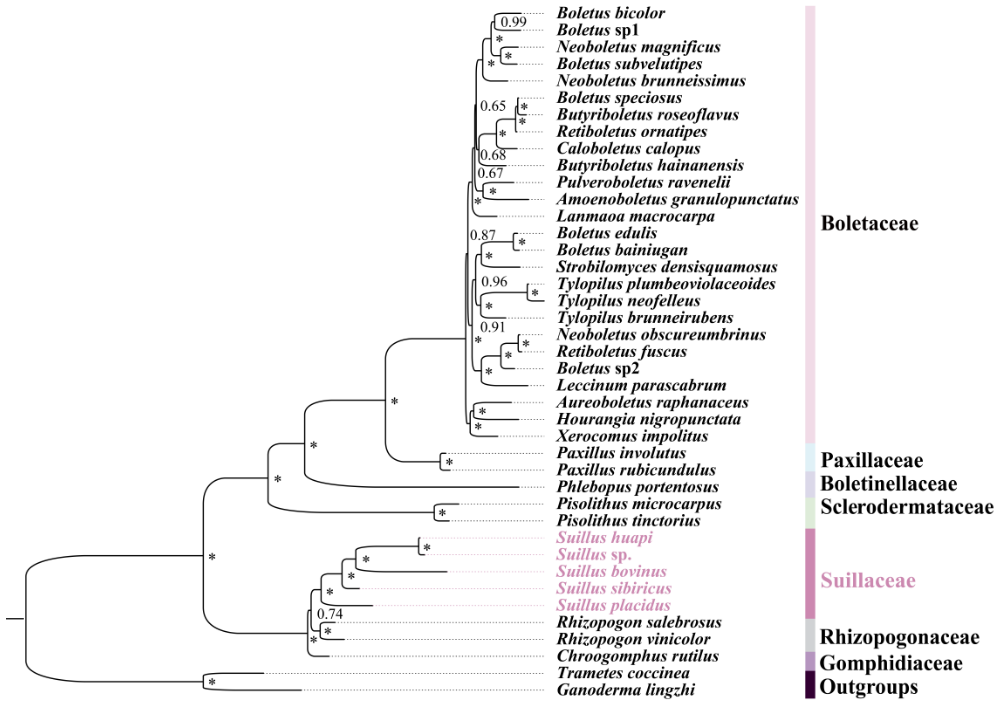

Figure S5. Phylogenetic relationship of Boletales based on PCG12 using MrBayes. The symbols * indicate that the posterior probability of this node is 1. Five newly sequenced mitogenomes of *Suillus* were highlighted in lavender.

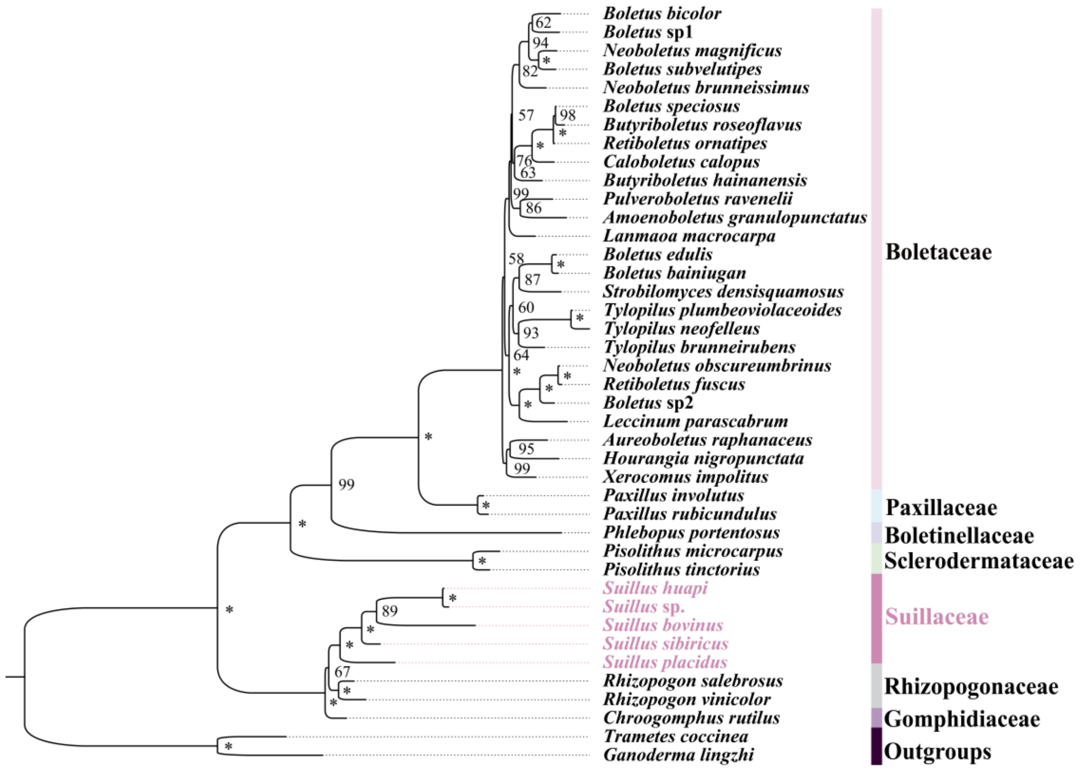

Figure S6. Phylogenetic relationship of Boletales based on PCG12 using IQ-TREE. The symbols * indicate that BS of this node is 100. Five newly sequenced mitogenomes of *Suillus* were highlighted in lavender.

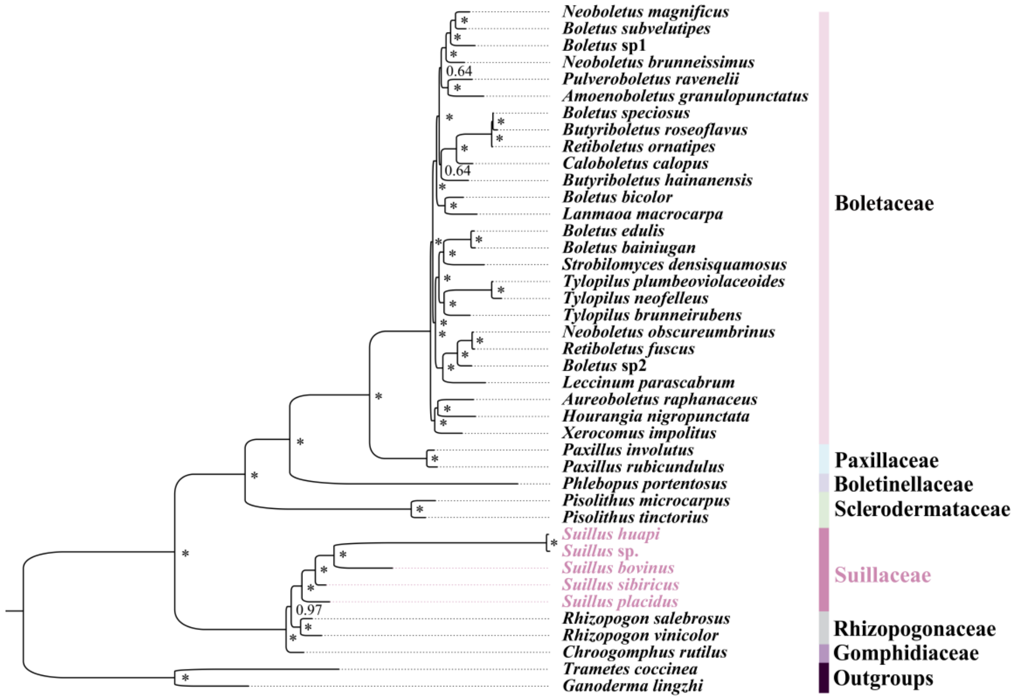

Figure S7. Phylogenetic relationship of Boletales based on PCG12RNA using MrBayes. The symbols * indicate that the posterior probability of this node is 1. Five newly sequenced mitogenomes of *Suillus* were highlighted in lavender.

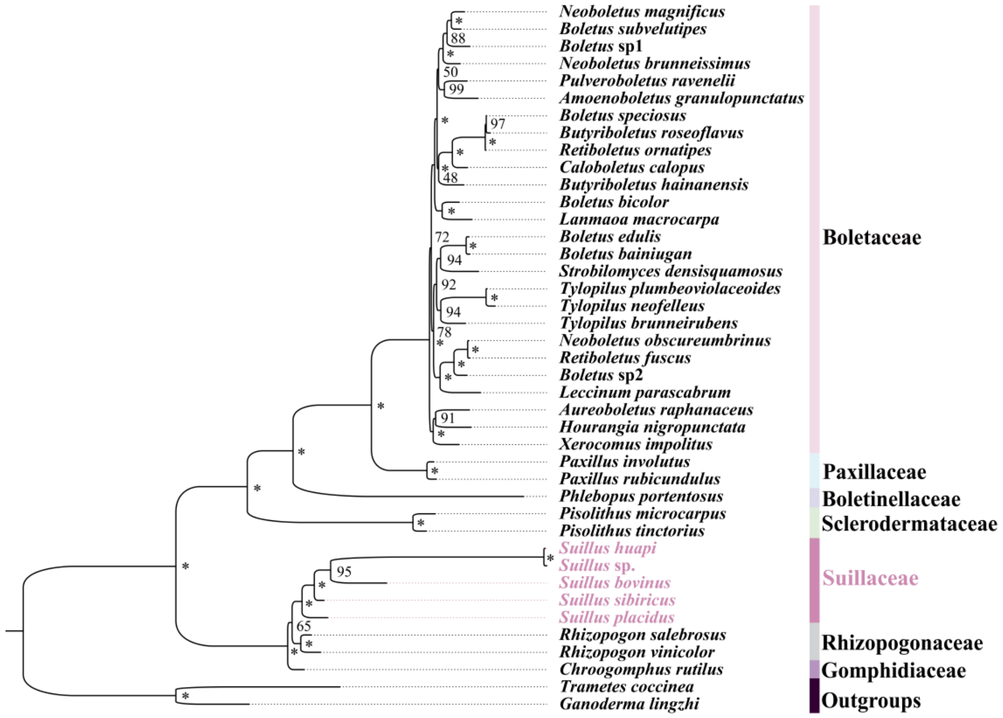

Figure S8. Phylogenetic relationship of Boletales based on PCG12RNA using IQ-TREE. The symbols * indicate that BS of this node is 100. Five newly sequenced mitogenomes of *Suillus* were highlighted in lavender.

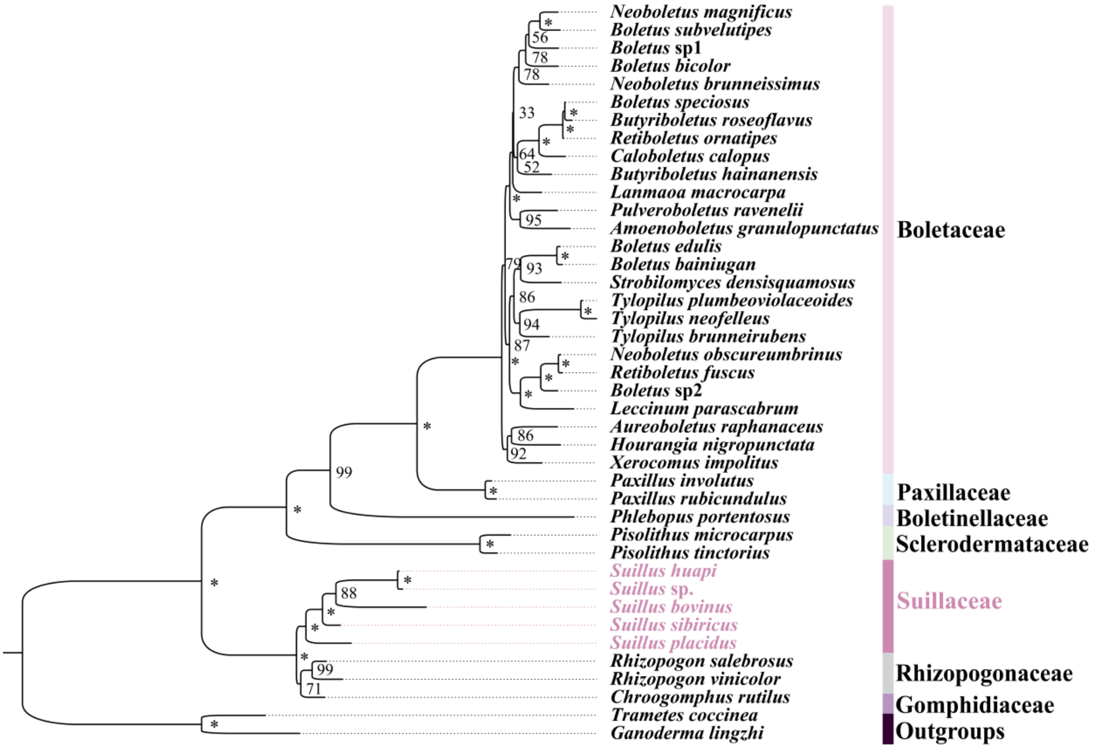

Figure S9. Phylogenetic relationship of Boletales based on AA using IQ-TREE. The symbols * indicate that BS of this node is 100. Five newly sequenced mitogenomes of *Suillus* were highlighted in lavender.

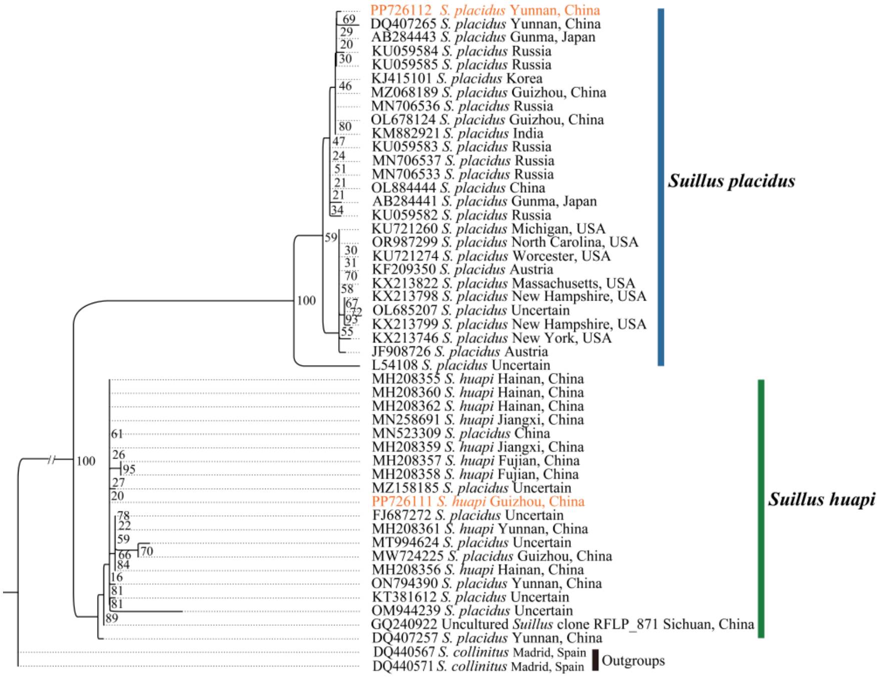

Figure S10. Phylogenetic relationship of *Suillus placidus* and *Suillus huapi* inferred from an *ITS* dataset using maximum likelihood. Our newly sequenced molecular fragments were highlighted in orange.
